# Non-linear relationship between built environment and non-motorized travel efficiency under the traffic micro-circulation model

**DOI:** 10.1371/journal.pone.0314050

**Published:** 2025-01-30

**Authors:** Xiaoyuan Dong, Lining Wang, Sen Du, Bicheng Qian, Jiaxin Wang

**Affiliations:** School of Architecture and Urban Planning, Lanzhou Jiaotong University, Gansu, Lanzhou, China; Sakarya University: Sakarya Universitesi, TÜRKIYE

## Abstract

The built environment is an important determinant of travel demand and mode choice. Studying the relationship between the built environment and transportation usage can support and assist traffic policy interventions. Previous studies often assumed that this relationship is linear; however, the impact of the built environment on non-motorized travel efficiency may be more complex than the typically modeled linear relationships. This paper focuses on the core area of Chengguan District in Lanzhou City, utilizing multi-source big data including POI, OpenStreetMap, street view images, and built environment data. Using ArcGIS spatial analysis tools combined with the Extreme Gradient Boosting (XGBoost) model, we analyze the non-linear influence mechanisms and threshold effects of the built environment on non-motorized travel efficiency and establish a ranking of the relative importance of all built environment factors. The results indicate that factors such as the branch road/street, land-use mix, land-use density, neighborhood entrance/exit density, bus station density, and dead-end-roads density are key influences on non-motorized travel efficiency. Additionally, based on the non-linear thresholds presented in the partial dependence plots for built environment factors, this paper proposes optimization strategies for small-scale road network patterns, mixed land use, and bus-friendly environments, providing effective threshold ranges and decision-making references for urban planning and traffic management.

## 1. Introduction

The disorderly expansion of cities and excessive reliance on motorization have brought numerous problems to urban transportation. Coupled with the "car-centric" mindset, this has led to a series of negative externalities such as traffic congestion, conflicts between motorized and non-motorized transport, and environmental pollution, thereby exacerbating the contradictions among population, transportation, and urban land use [1–3]. The micro-circulation system is an important means to address urban traffic congestion, save land resources, and facilitate scientific road network planning [4–6]. At the same time, non-motorized travel, as a green and healthy mode of transportation, is an effective way to enhance residents’ travel efficiency and alleviate urban traffic issues [7]. However, in many existing cities in the country, the built environment prioritizes motor vehicle operation, resulting in inadequate support for non-motorized travel activities. This leads to a lack of safe and comfortable operating space for non-motorized vehicles, indicating that the non-motorized travel environment needs further improvement [8]. From the perspective of urban planning and design, the relationship between the built environment and urban residents’ travel has always been a focal point in urban design and transportation planning [9–11]. Many urban design strategies proposed today, such as open blocks and multifunctional streets, aim to better promote slow traffic [12]. Research on the factors influencing non-motorized travel efficiency often focuses more on built environment factors [13–15]. Therefore, a comprehensive understanding of the intrinsic relationship between non-motorized travel and built environment factors can better guide and enhance the development of slow traffic and micro-circulation through the built environment, providing improved support for non-motorized travel activities in urban areas.

Guiding transportation through the built environment has become a trend; however, research by domestic scholars is still in its early stages and presents several shortcomings [16,17]:①In terms of research content, there is relatively less research in China regarding the relationship between non-motorized travel behaviors and the built environment compared to international studies, especially concerning how the built environment affects non-motorized travel efficiency. Existing studies mainly focus on non-motorized travel preferences and the relationship between travel preferences and environmental factors [18,19]. ②In terms of research methods and strategies, previous studies on the impact of the built environment on transportation have largely employed linear models, such as linear regression, structural equation modeling, and geographically weighted regression [20–23]. These approaches obscure the existing non-linear relationships and threshold effects. Additionally, past research has often remained at the level of descriptive statistics and model construction, without proposing corresponding threshold ranges and planning strategies based on the mechanisms of influencing factors.

Based on this, the paper approaches the study from the perspective of promoting traffic micro-circulation in the central urban area, specifically examining certain high-congestion zones in the Chengguan District of Lanzhou City to explore the non-linear relationship between the built environment and non-motorized travel efficiency. To better guide planning practices, empirical research is required to test these thresholds and examine their effectiveness. Lanzhou, a typical valley-type city, serves as the chosen research subject. Its unique geographical feature of being " two mountains and one valley " [24] limits the available land scale and the integrity of the transportation network, exacerbating transportation issues and resulting in a scenario characterized by "crowding in the east-west direction and poor connectivity in the north-south direction." By gaining a deep understanding of the relationship between the built environment factors and non-motorized travel efficiency in Lanzhou, this study aims to derive effective threshold ranges and planning recommendations. This can provide better support for urban residents’ non-motorized travel and offer insights and references for other valley-type cities in China regarding the creation of a conducive non-motorized travel environment and the improvement of traffic micro-circulation efficiency.

The rest of this paper is organized as follows: In Section 2, a review of the research on the attributes of the built environment and travel behavior, including their non-linear relationships, is presented. This section also compares the models and methods used in relevant studies. Section 3 introduces the data and modeling methods employed in this research. In Section 4, the relative importance and non-linear relationships derived from the quantitative analysis are discussed, along with an explanation and interpretation of the results, and the policy implications are provided. Finally, Section 5 summarizes the main findings and presents future outlooks for further research.

## 2. Literature review

### 2.1 The relationship between the built environment and travel behavior

The built environment is a crucial determinant of travel demand and mode choice [25], with numerous studies revealing its significant role in influencing transportation behaviors [26]. The built environment refers to various buildings and spaces that have been constructed or modified by humans, particularly those environments that can be altered through policies and human actions. It is a combination of a series of elements related to land use, transportation systems, and urban design. The "5D" model—comprising Density, Diversity, Design, Destination Accessibility, and Distance to Transit—has laid the foundation for many subsequent studies on the "built environment-travel behavior" relationship [27]. Research indicates that in rural areas of China, built environment factors significantly affect transportation modes. Elements such as building density, road density, bus stops, and land use patterns play varying roles in determining residents’ travel choices. Proper land use planning and traffic network design can effectively shape green travel and sustainable transport development in rural regions [9,28]. In studies utilizing big data on residential travel patterns in large housing complexes in Shenzhen and Shanghai, it was found that job density and proximity to bus stops positively influenced residents’ travel behaviors, while residential density and distance to subway stations had negative effects. Optimizing built environments—through friendly designs of commercial facilities and road intersections, as well as providing more sports facilities—can enhance residents’ willingness to engage in active travel [11,29]. The impact of the built environment on travel modes varies across different demographics. For instance, a study on gender differences in active travel among the elderly found that population density contributed more significantly to active travel among women, whereas the distance to cafes or leisure venues affected men’s active travel time more substantially [30]. Research exploring the human-scale quality of street environments revealed significant associations between street conditions and children’s likelihood of walking to school [31]. Public transport patterns are also influenced by the built environment. In Brisbane, Australia, transit-oriented development (TOD) communities showed significantly higher public transport usage [32]. Furthermore, findings indicated that the public transport usage among the elderly is affected by built environment factors such as intersection density, road network density, and the density of public service facilities [33]. Studies analyzing ride-hailing services using order data have also highlighted the spatial and temporal variations in demand relative to built environment factors [34]. Different elements—such as land use diversity, job-residential ratios, road attributes, and transport accessibility—are closely linked to ride-hailing demand [35,36]. Additionally, research on shared bicycle usage has shown that various built environment factors exhibit spatial differentiation in their effects on bike-sharing practices [23]. For example, a study on shared bicycle usage in Boston found a significant positive correlation between riding frequency and factors like neighborhood population, road network density, and subway accessibility [37]. Similarly, research in Xiamen indicated that company points of interest (POIs), building density, and intersection counts were positively correlated with the rate of bike returns, while spatial enclosure and proximity to the nearest subway station had negative correlations [38]. Overall, these findings underscore the complex interactions between built environment characteristics and various travel behaviors across different populations and contexts.

### 2.2 The built environment and nonlinear relationships

Many studies have found that certain attributes of the built environment have nonlinear and threshold effects on transportation activities [25,39]. For example, the impact of the built environment on shared bicycle usage may be more complex than the linear relationships typically modeled [40]. A study surveyed 18 communities in Guangzhou, China, to explore the nonlinear relationship between the built environment of residential areas and workplaces and carbon dioxide commuting emissions, indicating that the built environment at the workplace has a greater impact on carbon dioxide emissions than that of the residence [41]. When investigating the nonlinear relationship between the built environment and travel distance using data from Oslo, both models employed showed a significant nonlinear relationship [42]. In Shanghai, the characteristics of the built environment surrounding workplaces exhibited clear linear relationships and threshold effects related to adults’ commuting and leisure walking [43]. In the U.S. Twin Cities region, there is a general threshold effect between driving, public transit, and active travel distances relative to the built environment. The study identified common thresholds for architectural features associated with three types of travel distances and suggested that planners allocate limited resources effectively across different scales of planning initiatives [44]. Research into the relationship between the built environment and electric scooter sharing passengers revealed that previous linear studies might lead to inaccurate passenger forecasts and ineffective policies. Understanding the nonlinear relationship between the built environment and electric scooter usage can help planners identify high-demand areas and design effective investment plans to promote micro-mobility [45]. Additionally, the built environment in the 15-minute living circle should possess daily commuting attractions to "retain" as many daily outings as possible within the area. Some studies indicate that there is spatial heterogeneity in the nonlinear influence of "commuting retention rate" and the built environment [16]. An empirical study of urban rail transit travel in Chengdu, China, found that road network density, floor area ratio, and traffic facilities all have a nonlinear positive impact on passenger flow, while housing prices and distances to the CBD exhibited nonlinear relationships of "convex" and "concave" shapes, respectively [46].

### 2.3 Methods used in related research

In recent years, the application of big data has overcome the limitations of traditional data collection methods, particularly as open-source urban big data provides new perspectives and methods for studying urban residents’ travel patterns [47,48]. In terms of data collection related to the built environment, the development of online electronic maps has led many scholars to apply point of interest (POI) data in urban planning research. The processing and utilization of POI data facilitate a more detailed and comprehensive understanding of urban spatial structures, expanding the sources of data available for studying the built environment [49,50].

Regarding research methodologies, most past studies have used statistical modeling to explore the relationship between the built environment and travel behavior, commonly employing several widely-used models: Geographic Weighted Regression (GWR), Ordinary Least Squares (OLS), and discrete choice models such as Logit and Probit models. When examining the relationship between the built environment and subway passenger volume, the GWR model can calibrate the spatial weight matrix by calculating grid distances, thereby accurately representing the relationships between stations [22]. Additionally, some studies have utilized both OLS and GWR models to investigate how different holidays affect the relationship between the built environment and subway passenger volume, comparing the varying impacts of these holidays to aid in predicting subway usage and ensuring fairer and more efficient resource allocation [51]. When studying travel behavior, researchers often use the Multinomial Logit (MNL) model to analyze economic factors and assess travel behavior choices [52,53].

With the deepening of research, artificial intelligence, machine learning, and deep learning techniques derived from machine learning have begun to be applied to urban issues. Many scholars are using machine learning models to explore nonlinear relationships. Relevant studies have utilized machine learning to examine the nonlinear relationships between rail transit travel and the built environment [46,54,55], between shared cars and bicycles and the built environment [56–60], and between residents’ travel modes and the built environment [13,61,62]. Among these, the Extreme Gradient Boosting (XGBoost) algorithm is effective in revealing nonlinear relationships; it is similar to Gradient Boosting Decision Trees (GBDT) but offers insights into the relative contributions of features and nonlinear relationships [58]. Due to its strong predictive performance and reliable identification of variable importance, this algorithm has gradually been applied in the transportation field in recent years [63].

### 2.4 Domestic and international research summary

The above literature has comprehensively discussed the relationship between the built environment and travel behavior, non-linear effects, and the methods used in related research. A review of existing literature reveals that the built environment is a significant influencing factor on travel demand and mode choice, with its effects being multidimensional and complex. In particular, the introduction of the "5D" model has provided an important theoretical framework for subsequent studies. Furthermore, research indicates that the characteristics of the built environment can influence travel patterns in various ways across different regions and populations. The diversity of existing research methods, including quantitative analysis and qualitative studies, offers rich perspectives for understanding this relationship. However, these studies often occur independently and lack an overarching framework. Therefore, future research should focus on integrating these fields to explore the interaction between the built environment and travel behavior from a more systematic perspective, thereby providing stronger support for urban planning and transportation policy development.

## 3. Method

### 3.1 Data and variables

#### 3.1.1 Research area selection and basic unit delineation

Lanzhou is a typical river valley city, characterized by a narrow topography defined as "two mountains flanking a valley." This geographical limitation restricts the integrity of the city’s road network. Compared to other similarly sized plain cities, the internal traffic in Lanzhou primarily runs along an east-west axis, concentrating most of the city’s traffic volume in this direction. As a result, there is a situation of "congestion in the east-west direction and poor flow in the north-south direction." If urban planning is not adequately rationalized, the complex contradictions between land use and transportation functions will become more pronounced [24]. The most representative road segments in the city are Xijin Road, Binhai Road, and Baiyin Road. These three roads are located in the narrowest part of the city and serve as main east-west thoroughfares, accommodating both through traffic and local traffic. Xijin East Road and Binhai Central Road constitute the "waist" section of Lanzhou, where, during peak periods, these east-west main roads can experience complete traffic jams, making traffic congestion a significant bottleneck affecting urban development. Chengguan District, as the central old town of Lanzhou, accounts for more than 50% of the city’s employment opportunities and population. The excessive concentration of these two factors leads to the greatest attraction of Chengguan District towards the other three districts in the center, while also imposing greater pressure on its traffic, resulting in a relatively high probability of congestion and saturation [64].

**Fig 1:**
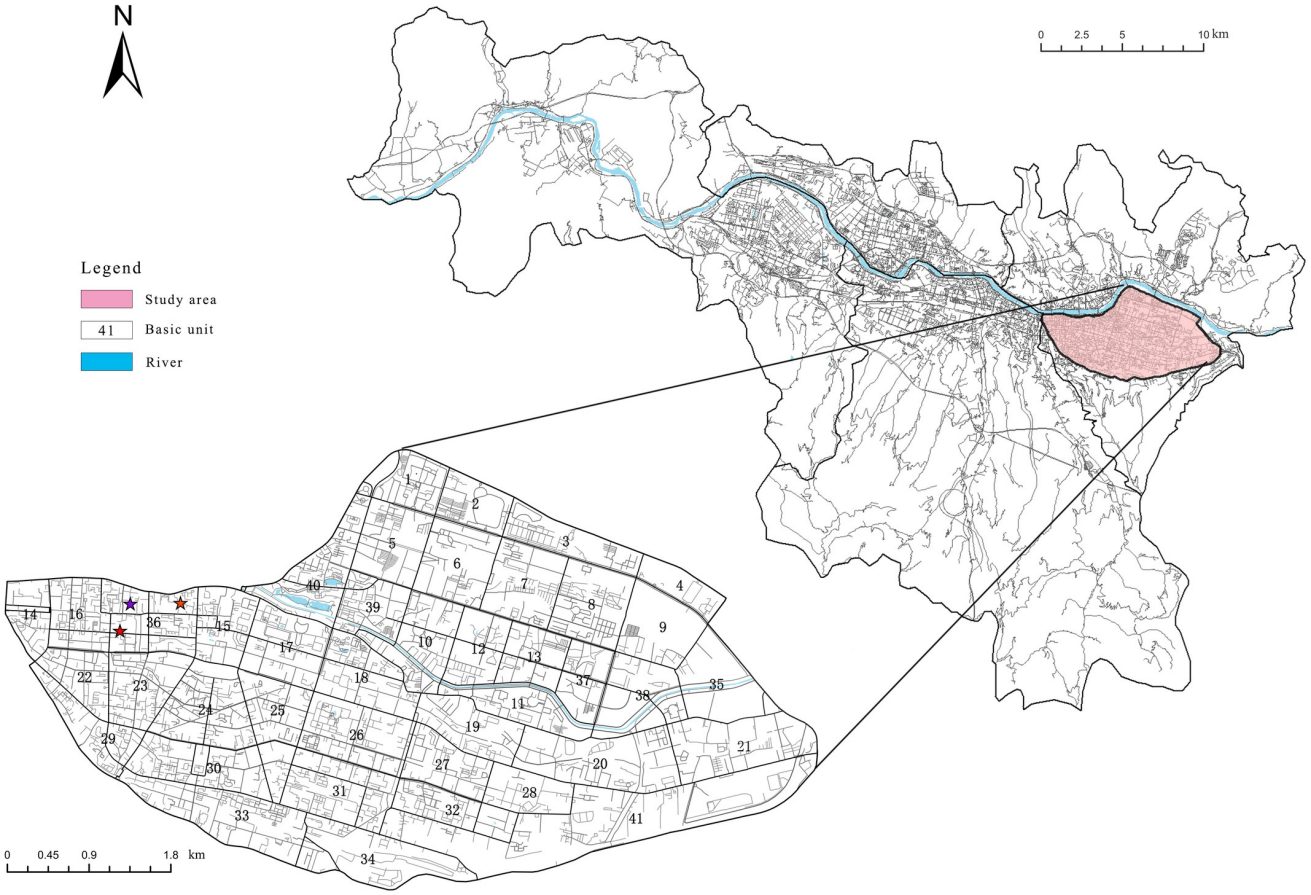
Selection of research scope.

This study selects the most typical densely populated traffic areas within Chengguan District of Lanzhou as the research subject. Due to the constraints imposed by the river and other influences, the research scope is defined by the boundaries of south of the Yellow River, north of Nanhuan East Road, east of Cuiyingmen Road, and west of Donggang Interchange (Fig 1). The study will conduct an in-depth examination of the microcirculation organization of traffic within this area and the nonlinear relationship between the built environment and non-motorized travel efficiency.

In order to facilitate the calculation of traffic microcirculation efficiency and to analyze the nonlinear relationship between the built environment and the efficiency of non-motorized transportation, it is necessary to divide the study area into basic units. Based on the definition of traffic microcirculation (which refers to the network of areas composed of secondary roads, branches, and some local streets outside the main urban thoroughfares) [6], and in conjunction with the methods for defining research units from the study on traffic microcirculation operation evaluation and optimization in the ancient urban area of Suzhou [65], this research will use primary and secondary arterial roads as the first-level linear division factors, while incorporating some branch roads as secondary linear factors. Additionally, by reasonably controlling the area of the basic units and other factors, the study area will ultimately be divided into 41 basic units. An in-depth study will then be conducted on the operating conditions of non-motorized vehicles and the efficiency of traffic microcirculation within these 41 basic units.

#### 3.1.2 data sources

1. Time-density data

Time density data, as the dependent variable in this study, is primarily obtained based on the 41 defined microcirculation basic units in the analysis of non-motorized transportation efficiency. This involves acquiring the location data of the geometric center points and edge exit points of the units. The route calculation function of Baidu Maps is utilized to obtain OD lines and calculate the path length. Additionally, by simulating the optimal travel routes using Baidu Maps’ best path feature, the optimal travel routes from each starting point to the destination are determined. Data collection was conducted from 7:00 to 18:00 on June 25, 2023, calculating the travel time for non-motorized vehicles every 30 minutes on both weekdays and weekends. Finally, ArcGIS is used to derive the average time density within each traffic microcirculation unit across multiple paths, multiple measurement nodes, and different time periods (specific calculations are shown in Eq 1).


$$Y=\frac{\sum_{i=1}^{n}\frac{t_{n}}{n}}{m}$$ (1)

**Fig 2:**
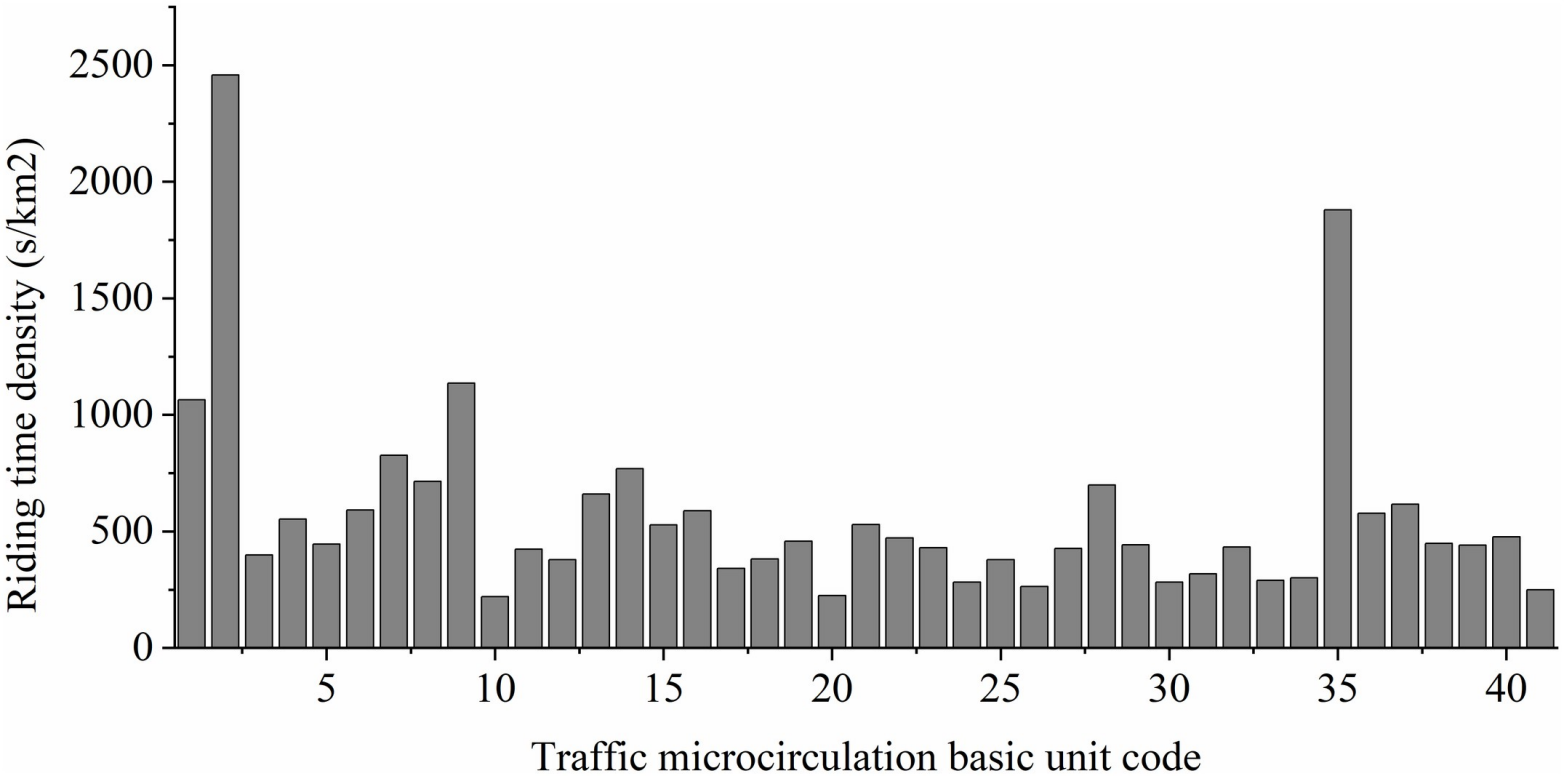
Non-motorized travel time density.

In the above equation, Y represents the time density of non-motorized vehicle operations, which is inversely proportional to the efficiency of non-motorized transportation. Here, n denotes the number of paths within the block, t refers to the travel time required for non-motorized vehicles along a specific path during a certain time period, and m represents the area of the traffic microcirculation basic unit (Fig 2).

2. Built environmental element data

Traffic microcirculation elements are generally based on road infrastructure, which not only includes the roads themselves but also encompasses various associated traffic organizations and facilities, as well as spatial elements related to traffic in both two-dimensional and three-dimensional contexts [66,67]. Through a thorough review and analysis of a substantial amount of relevant literature, several factors influencing non-motorized transportation within the built environment have been identified, forming the specific connotation of the built environment discussed in this paper. Based on the analysis and summary, this paper will describe the connotation of the built environment from five aspects: point elements, line elements, area elements, spatial elements, and organizational elements [68]. These elements include various variables such as road design, bus stops, public service systems, land use types, and street ambiance, as illustrated in Table 1.

**Table 1:** Traffic microcirculation elements table.

Microcirculation elements	Variables (indicators)	Description	Source	Sign
Point elements	Intersection Density	The ratio of the number of intersections in the basic unit to the size of the area	Open Street Map /Poi Collection Platform	x1
	Dead-End-Roads Density	The ratio of the number of dead-end-roads in the basic unit to the size of the area		x2
	T-Intersections Density	The ratio of the number of T-intersections in the basic unit to the size of the area		x3
	Branch-to-Trunk Interface Density	The ratio of the number of Branch-to-Trunk Interface in the basic unit to the size of the area		x4
	Neighborhood Entrance/Exit Density	The ratio of the number of neighborhood entrances/exits in the basic unit to the size of the area		x5
	Bus Station Density	The ratio of the number of bus stations in the basic unit to the size of the area		x6
	Public Service Facilities Density-Education	The ratio of the number of educational POIs in the basic unit to the size of the area		x7
	Public Service Facilities Density-Medical	The ratio of the number of medical POIs in the basic unit to the size of the area		x8
Line elements	Branch Line Density	The ratio of branch road length in the basic unit to the size of the area	Open Street Map	x9
	Street Line Density	The ratio of street length in the basic unit to the size of the area		x10
	Bus Routes Density	The ratio of bus route length in the basic unit to the size of the area		x11
	Branch Road/Street	The ratio of the Branch in the basic unit to the size of the area		x12
Surface elements	Land-Use Mix	Mixed entropy of land use	Lanzhou Natural Resources and Planning Bureau	x13
	Land-Use Density	The ratio of the number of parcels to the total area in the basic unit		x14
	Average Plot Size	Average plot size in the basic unit		x15
	Block Fragmentation Degree	Important features used to quantify the fragmentation of plots		x16
Spatial elements	Green Viewing Rate	The average percentage of green plant pixels at each streetscape sampling point in the unit	Baidu Street View Platform	x17
	Street Safety Degree	Average percentage of pixels of street furniture such as fences, street lights, etc. for each streetscape sampling point within the unit		x18
Organizational elements	Percentage of One-way Lanes	The ratio of one-way lane length to all lane lengths in the base unit	Baidu Map Open Platform	x19

The data required for the research includes urban road network data, point of interest (POI) data, land use data, and street view data. The point and line element data are primarily sourced from the OpenStreetMap open-source platform and POI data collection platforms. The area element data is mainly obtained from the Lanzhou City Planning and Natural Resources Bureau. Spatial element data is acquired by recognizing street view images through deep learning techniques, utilizing image semantic segmentation software based on fully convolutional neural networks to extract the number of pixels identified as vegetation or sky in each street panorama. The organizational element data is mainly obtained from the Baidu Street View platform and the Baidu Map Open Platform. After obtaining the relevant data from the open-source platforms, an assessment of data availability was conducted, followed by data cleaning and classification. The cleaned data were then imported into ArcGIS for error checking and density calculation. Subsequently, spatial feature analysis was performed on each basic unit to obtain the density data for various elements.

3. Variance inflation factor test

To ensure the selected variables are valid, a variance inflation factor (VIF) test was conducted first, excluding any variables with a VIF greater than 10. After a series of statistical analyses and screenings, two variables—intersection density (x1) and street network density (x10)—were excluded, resulting in the final valid variables, as shown in Table 2.

**Table 2:** Variance inflation factor test table.

Variables (indicators)	Sign	Non-normalized coefficients		Standardized coefficient	T	Significance	Covariance diagnosis	
		Regression coefficient	Standard error	Regression coefficient			Allowance	VIF
Dead-End-Roads Density	x2	3.831	6.147	0.173	0.623	0.539	0.245	4.089
T-Intersections Density	x3	0.373	3.194	0.034	0.117	0.908	0.219	4.57
Branch-to-Trunk Interface Density	x4	-6.249	15.298	-0.107	-0.409	0.687	0.275	3.633
Neighborhood Entrance/Exit Density	x5	-28.155	15.887	-0.309	-1.772	0.09	0.623	1.606
Bus Station Density	x6	-0.499	22.636	-0.005	-0.022	0.983	0.409	2.446
Public Service Facilities Density-Education	x7	-12.818	23.137	-0.117	-0.554	0.585	0.422	2.368
Public Service Facilities Density-Medical	x8	-25.075	98.739	-0.041	-0.254	0.802	0.722	1.385
Branch Line Density	x9	-0.897	49.306	-0.004	-0.018	0.986	0.332	3.014
Bus Routes Density	x11	31.142	25.562	0.232	1.218	0.235	0.52	1.922
Branch Road/Street	x12	233.36	173.545	0.313	1.345	0.192	0.349	2.868
Land-Use Mix	x13	-695.168	534.188	-0.263	-1.301	0.206	0.463	2.158
Land-Use Density	x14	11.167	21.21	0.189	0.526	0.604	0.147	6.793
Average Plot Size	x15	4741.865	3441.153	0.435	1.378	0.181	0.19	5.277
Block Fragmentation Degree	x16	112.218	792.821	0.025	0.142	0.889	0.582	1.717
Green Viewing Rate	x17	-1005.939	1906.289	-0.118	-0.528	0.603	0.378	2.646
Street Safety Degree	x18	-2835.431	4082.929	-0.134	-0.694	0.494	0.508	1.969
Percentage of One-way Lanes	x19	-875.086	1108.404	-0.148	-0.79	0.438	0.539	1.856

### 3.2 Method

#### 3.2.1 Extreme Gradient Boosting Model (XGBoost)

This study employs the Extreme Gradient Boosting (XGBoost) model for the analysis of nonlinear relationships. XGBoost is one of the most advanced machine learning algorithms available today and is an ensemble method that combines multiple weak classifiers to build regression and classification models [69]. It predicts data by constructing multiple subtrees and consolidates the predictions from all subtrees to obtain the final prediction result [70]. The ensemble model of XGBoost trees is as follows:

$${\hat{y}}_{i}=\overset{K}{\underset{k}{\sum }}f_{k}(x_{i}),f_{k}\in R$$ (2)

Where ${\hat{y}}_{i}$ is the predicted value of thmodel for the ith sample, K is the set of subtrees constructed by the model, is the correlation mapping relationship between the structure and weights of the kth tree in the set of subtrees, is the ith input vector, and R denotes the set of subtrees.

The objective function of XGBoost consists of two parts: the error value between the predicted and true values of the model, and a regular term that controls the complexity of the model, as shown in the following formula:

$$Y=\overset{n}{\underset{i=1}{\sum }}l(y_{i},{\hat{y}}_{i})+\overset{K}{\underset{k}{\sum }}\Omega (f_{k})$$ (3)

here $\sum_{i=1}^{n}l(y_{i},{\hat{y}}_{i})$ denotes the difference (error) between the predicted and true values of the model, and $\sum_{k}^{K}\Omega (f_{k})$ is the complexity of the tree model (i.e., the canonical term controlling the complexity of the model), which prevents the model from being overfitted, as illustrated in the following formula:

$$\Omega (f_{k})=\gamma M+\frac{1}{2}\lambda \overset{M}{\underset{j=1}{\sum }}\omega_{i}^{2}$$ (4)

Where γ, λ denote the penalty coefficients, M is the number of leaf nodes and ω is the fraction of leaf nodes.

The parameters in the tree-integrated model in Eq (2) contain unknowns that cannot be optimized in Euclidean space using traditional optimization methods, assuming that ${\hat{y}}_{i}^{\left(t\right)}$ is the prediction of the ith instance in the tth iteration, and f_t_ is added to minimize the following objective:

$$Y^{\left(t\right)}=\overset{n}{\underset{i=1}{\sum }}l(y_{i},{\hat{y}}_{i}^{\left(t-1\right)}+f_{t}(x_{i}))+\Omega (f_{k})$$ (5)

By adding the f_t_ that can best improve the model to achieve the goal of optimizing the function. In general, second-order approximations can quickly optimize the objective. The objective function is transformed by the second-order Taylor’s formula:

$$Y^{\left(t\right)}≅\overset{n}{\underset{i=1}{\sum }}[l(y_{i},{\hat{y}}^{\left(t-1\right)})+g_{i}f_{t}(x_{i})+\frac{1}{2}h_{i}f_{t}^{2}(x_{i})]+\Omega (f_{k})$$ (6)

Where $g_{i}=\partial_{{\hat{y}}_{i}^{\left(t-1\right)}}l(y_{i},{\hat{y}}^{\left(t-1\right)})$ and $h_{i}=\partial_{{\hat{y}}_{i}^{\left(t-1\right)}}^{2}\;l(y_{i},{\hat{y}}^{\left(t-1\right)})$ are the first-order and second-order gradient statistics of the loss function, respectively, and the removal of the constant term yields the final simplified XGBoost objective function:

$${\tilde{Y}}^{\left(t\right)}=\overset{n}{\underset{i=1}{\sum }}[g_{i}f_{t}(x_{i})+\frac{1}{2}h_{i}f_{t}^{2}(x_{i})]+\;\Omega (f_{k})$$ (7)

XGBoost has good modeling ability for the nonlinear characteristics of various types of data. Compared with neural networks, random forests, etc., XGBoost performs better and has higher fitting accuracy [63].

It has many advantages [69]:

①Predicted data is relatively accurate.②It has a strong acceptance of missing data.③It can predict the relationship between the independent and dependent variables without predetermining the relationship in advance (e.g., linear). Thus it can better detect the existing nonlinear relationship. At the same time, it has some disadvantages [63]. For example, it cannot perform significance tests, nor can it give the coefficients of the independent variables and their confidence intervals, but it can reflect the relative importance of the independent variables, and it can also visualize the relationship between the independent variables and the dependent variable, which can make up for the above shortcomings.

#### 3.2.2 Model construction and accuracy verification

The model construction process is divided into 4 steps. In the first step, in order to ensure the interpretability of the model, the variance inflation factor (VIF) is applied to screen the independent variables, and the variables with VIF>10 are excluded to ensure that there is no covariance among the independent variables; in the second step, 80% of the samples are randomly selected as the training set, and the remaining 20% is used as the test set for evaluating the performance of the model; in the third step, in order to optimize the quasi-mingled rate of the model and to avoid under- or overfitting, the adjust the hyper-parameters of the model, including the learning rate, the maximum depth of the tree, and so on. Finally, the accuracy, generalization and overfitting of the model are evaluated.

In this paper, we adopt the "hyperparameter grid search method" to determine the optimal hyperparameters, which is to train all the listed parameter combinations cyclically until the optimal parameter combination is found. In this paper, two hyperparameters are selected and their values are restricted: the possible values of learning rate are set to (0.01, 0.015, 0.025, 0.05, 0.1) and the possible values of maximum tree depth are set to (3, 4, 5, 6, 7). The final results show that the model performance is optimal when the learning rate is 0.05 and the maximum tree depth is 7. In this paper, the R2 of XGBoost model is 0.71, and the R2 of OLS model is 0.63, so it can be seen that XGBoost model has better explanatory power.

## 4. Results and discussion

### 4.1 The relative importance of built environment elements

Relative importance is one of the most commonly used analytical methods in machine learning. During the process of constructing decision trees in the Extreme Gradient Boosting model, each independent variable has a certain probability of being selected to split the data, essentially dividing the data space into two parts [69]. In essence, relative importance refers to the proportion of the number of times a particular variable is chosen during the iterative construction of decision trees compared to the total number of selections made for all independent variables. Relative importance is typically expressed as a percentage, with the sum of the importance of all independent variables equal to 1 (or 100%). It can accurately represent the contribution of each independent variable to the prediction of the dependent variable. Table 3. displays the relative importance and ranking of all built environment independent variables.

**Table 3:** Relative importance of independent variables.

Variables (indicators)	Sign	Relative importance	Rankings
Branch Road/Street	x12	36.22%	1
Land-Use Mix	x13	16.62%	2
Land-Use Density	x14	12.57%	3
Neighborhood Entrance/Exit Density	x5	9.74%	4
Bus Station Density	x6	3.34%	5
Dead-End-Roads Density	x2	2.88%	6
Average Plot Size	x15	2.74%	7
Green Viewing Rate	x17	2.67%	8
Bus Routes Density	x11	2.62%	9
Street Safety Degree	x18	2.14%	10
Block Fragmentation Degree	x16	2.03%	11
Percentage of One-way Lanes	x19	2.02%	12
Branch Line Density	x9	1.80%	13
T-Intersections Density	x3	1.27%	14
Public Service Facilities Density-Education	x7	0.52%	15
Public Service Facilities Density-Medical	x8	0.50%	16
Branch-to-Trunk Interface Density	x4	0.33%	17

This paper selects the variables with the relative importance of the top 80% to explain. Among all the independent variables, the branch road/street (x12) has the highest influence weight, accounting for 36.22%, followed by the land-use mix (x13), accounting for 16.62%, followed by land-use density (x14), neighborhood entrance/exit density (x5), bus station density (x6), dead-end-roads density (x2), etc.

### 4.2 Non-linear relationship between key independent variables and non-motorized vehicle operation

**Fig 3:**
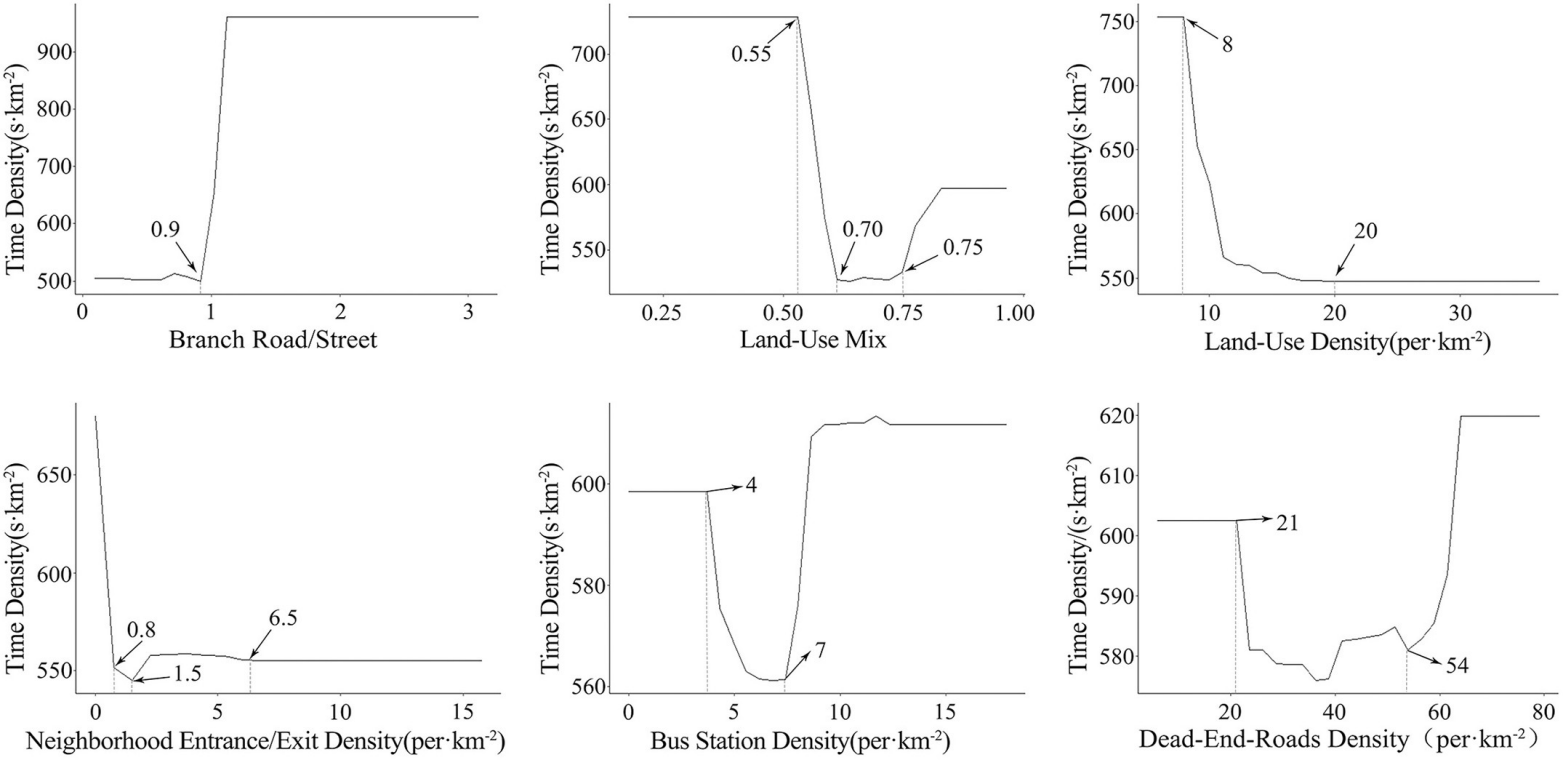
The nonlinear relationship between built environment elements and non-motorized travel efficiency.

Partial dependence plots are also a commonly used analytical method in machine learning. This method visualizes the relationship between the dependent variable and independent variables, thereby providing an intuitive representation of the actual effects of each independent variable on the dependent variable. Based on the previous analysis of the relative importance of various elements of the built environment, the top six ranked variables were selected to analyze the nonlinear relationship between the built environment and the efficiency of non-motorized travel. Fig 3 presents the partial dependence plots for the selected top six independent variables.

1. Non-linear relationship between branch road/street and non-motorized travel efficiency
  The branch road/street exhibits a positive nonlinear relationship with the travel time required for non-motorized vehicles, and there is a notable threshold effect. The branch/street represents the ratio of the length of branch streets to the number of street segments; a higher ratio indicates a lower density of street segments relative to the length of branch streets, which corresponds to poorer road accessibility. From the figure, it can be observed that when the ratio is between 0 and 0.9, the travel time required for non-motorized vehicles is relatively short, indicating overall high efficiency. However, once the ratio exceeds 0.9, the efficiency of non-motorized travel begins to decline sharply. This suggests that the construction of street segment density should be maintained within a reasonable range: the higher the density of street segments relative to the length of branch streets, the better the road accessibility, and consequently, the greater the support provided by the built environment for non-motorized vehicle operations.
2. Non-linear relationship between land-use mix and non-motorized travel efficiency
  The land-use mix exhibits a "U"-shaped nonlinear relationship with the travel time required for non-motorized vehicles. Within the basic unit of microcirculation, the land-use mix significantly influences the travel efficiency of non-motorized vehicles; its layout can substantially enhance the frequency and proportion of non-motorized trips to some extent. From the figure, it is evident that when the land-use mix is between 0 and 0.55, its impact on the travel efficiency of non-motorized vehicles is relatively low. However, once the degree exceeds 0.55, as the land-use mix increases, the travel time required for non-motorized vehicles decreases, and operational efficiency rises sharply. At a land-use mix of 0.70, non-motorized travel times are minimized, achieving the highest efficiency. Nevertheless, beyond a degree of 0.75, as the land-use mix continues to increase, the efficiency of non-motorized travel begins to decline. This indicates that while the land-use mix can enhance the efficiency of non-motorized travel within certain threshold limits, it does not necessarily lead to a fundamental reduction in traffic.
3. Nonlinear relationship between land-use density and non-motorized travel efficiency
  The land-use density exhibits a negative nonlinear relationship with the travel time required for non-motorized vehicles. When other influencing factors are held constant, lower land-use density indicates larger overall plot sizes, which exacerbate road congestion. In contrast, areas with high land-use density tend to have smoother traffic and higher accessibility, primarily facilitating walking or non-motorized travel. From the figure, it is evident that when the land-use density reaches 8 (units/km^2^), as the land-use density increases, the travel time required for non-motorized vehicles decreases, and operational efficiency rises sharply. Once the density reaches 20 (units/km^2^), the efficiency of non-motorized travel stabilizes, showing no further improvement with increased land-use density. Overall, areas with high land-use density tend to have more roads and shorter travel distances, resulting in higher efficiency for non-motorized travel. Conversely, low-density areas typically have longer travel distances, leading to lower efficiency for non-motorized modes.
4. Nonlinear relationship between neighborhood entrance/exit density and non-motorized travel efficiency
  The neighborhood entrance/exit density exhibits a negative nonlinear relationship with the travel time required for non-motorized vehicles. As indicated in the figure, with the increase in the density of entry and exit points within a basic unit, the travel time required for non-motorized vehicles decreases, leading to improved travel efficiency. When the neighborhood entrance/exit density exceeds 0.8 (units/km^2^), travel times start to decline further, reaching the highest operational efficiency at a density of 1.5 (units/km^2^). However, beyond a density of 6.5 (units/km^2^), the efficiency of non-motorized travel no longer improves with the increasing density of entry and exit points. A higher number of entry and exit points indicates greater connectivity within the basic unit, providing more route choices from central locations to peripheral intersections, which enhances the operational efficiency of non-motorized travel to some extent. Conversely, if the density becomes too high, it can lead to an increased number of intersections, causing delays in travel time and reducing overall travel efficiency.
5. Nonlinear relationship between bus station density and non-motorized travel efficiency
  The bus station density exhibits a "U"-shaped nonlinear relationship with the travel time required for non-motorized vehicles. When the bus station density is between 0 and 4 (units/km^2^), it has minimal impact on the efficiency of non-motorized travel. However, in the range of 4 to 7 (units/km^2^), as the bus station density increases, the travel time required for non-motorized vehicles decreases, and operational efficiency rises sharply. Beyond 7 (units/km^2^), higher bus station density leads to increased travel times and gradually reduced efficiency for non-motorized travel. In areas with low bus station density, the network hierarchy is often unclear, and routes can be convoluted, leading to longer travel times. As the bus station density increases, the demand for solutions to “last-mile” travel challenges also rises, prompting individuals to favor non-motorized transportation options. However, if the bus station density exceeds a certain threshold, the efficiency of non-motorized travel may decline due to excessive congestion and complexity. Therefore, the layout of bus stops should be maintained within a reasonable range to optimize non-motorized travel efficiency.
6. Nonlinear relationship between dead-end-roads density and non-motorized travel efficiency
  The dead-end-roads density exhibits a "U"-shaped nonlinear relationship with the travel time required for non-motorized vehicles. From the figure, it can be observed that when the dead-end-roads density is within the range of 21 to 54 (units/km^2^), the travel time for non-motorized vehicles is relatively short, and operational efficiency is comparatively high. Conversely, when the density is below 21 (units/km^2^) or above 54 (units/km^2^), the travel time increases, resulting in generally lower operational efficiency for non-motorized travel. dead-end-roads density reflects the accessibility and connectivity of road networks to some extent. When there are too few dead-end-roads within a basic unit, it indicates that the internal road network density may not be very high, leading to poor accessibility. On the other hand, an excessive number of dead-end-roads suggests inadequate road connectivity, indicating that the links between different road networks may be inefficiently connected. This situation ultimately reduces the operational efficiency of non-motorized vehicles. Therefore, maintaining an optimal dead-end-roads density is essential to support efficient non-motorized travel.

### 4.3 Policy implications

Through the study of the nonlinear relationship between the built environment and non-motorized vehicle travel in high-congestion areas of Chengguan District in Lanzhou, as well as the overall traffic microcirculation operational efficiency, effective thresholds and the following strategies for optimizing the built environment are provided.

(1) Small-scale road network pattern

The density of street networks and parcel scale are the primary factors influencing the efficiency of non-motorized travel and overall traffic microcirculation. An effective microcirculation network structure should ideally resemble a "pyramid," with appropriately sized parcels. However, research indicates that most basic units exhibit a "reverse triangle" or "spindle" shape in their road network structure, often featuring oversized parcels and a general lack of internal streets. In cases where the internal street network is insufficient or parcels are too large, a significant number of short-distance trips need to be redirected through sub-roads and higher-level roads, increasing detour times and exacerbating road congestion, which leads to local microcirculation inefficiencies. It is important to ensure that increases in road network density remain within reasonable thresholds; excessively dense networks can result in shorter distances between intersections and an increased number of them, further complicating traffic organization and management, ultimately reducing non-motorized travel efficiency.

Therefore, planning for road network density and parcel scale should integrate urban residential and employment distributions, spatial layouts, land use, and economic development. Attention should be given to enhancing the density of internal streets. For oversized blocks, it is advisable to open up internal roads and connect T-junctions and dead-end-roads within the sub-road network to improve street accessibility. In future planning cycles, it is recommended to incorporate the planning of sub-roads and lower-level road networks into the urban planning system, ensuring that street networks effectively facilitate microcirculation efficiency. Additionally, parcel scales should be taken into account by establishing road network planning based on defined parcel sizes to enhance the rational structural organization of road networks for smoother traffic microcirculation operations.

(2) Land-use mix

There exists a mutually reinforcing and constraining bidirectional relationship between urban land use and transportation. On one hand, the form of land use often serves as a source of traffic generation. Land use not only determines the occurrence, attraction, and mode choice of transportation but also establishes demand and supply patterns on a macro level. Therefore, different land use scenarios imply different transportation development models. On the other hand, the development of urban transportation can adversely affect land use and spatial structure because transportation can alter the accessibility of various regions, which in turn has a decisive impact on the scale, function, and intensity of land use. Generally speaking, areas with higher land density and functional diversity are better able to promote a balance between residential and employment options, significantly increasing the frequency of non-motorized and slow travel, thus facilitating traffic microcirculation to some extent.

Therefore, during the stages of urban master planning and detailed regulatory planning, it is essential to maintain a moderate balance among various types of land uses, such as residential, commercial, and public facilities, while altering single-purpose land use patterns. This approach enables residents to meet their daily living and shopping needs within appropriate ranges, achieving an efficient balance among diverse land uses. In the stage of detailed construction planning, emphasis should be placed on mixed arrangements of various business formats to foster a balance between residence and employment, improve commuting conditions, consider a multi-center development model, and change the existing separation of residence and employment in old urban areas, along with pendulum traffic patterns. This will help reduce car usage and commuting times for residents, promoting green commuting. In future developments, efforts should guide people towards adopting green transportation as the primary mode of travel, decreasing car usage rates, returning to a human-centered transportation philosophy, and facilitating the efficient operation of traffic microcirculation.

(3) Promoting Bus Friendliness

The unreasonable density and distribution of bus stops and routes can result in conventional bus services being confined to the main roads of the city, preventing them from penetrating into residential areas. As a result, travelers must walk a considerable distance to board a bus, which also leads to high redundancy rates for buses on major roads and an increased likelihood of bus bunching. This severely affects the quality and attractiveness of bus services and indirectly reduces the efficiency of non-motorized transportation. Moreover, as the old district of Lanzhou City, Chengguan District still requires improvements in its bus layout in several aspects, such as the selection of bus stop locations near intersections and the types of bus stops along certain segments. Research has identified many non-bay-style bus stops in the area, which do not fully utilize the potential road space, causing disruptions to vehicles and pedestrians traveling normally when buses pull into stops. Additionally, many bus stops are located upstream from intersections, and sometimes buses occupy motor vehicle lanes, resulting in delays for other non-bus vehicles. This, to some extent, reduces the operational efficiency of overall traffic microcirculation.

In future development and planning, it is important to coordinate the connection points between secondary roads and urban roads as well as the layout of urban rail transit stations to rationally arrange bus stops. It is recommended to consider using bus stops as the center point and walking and non-motorized travel distances as the radius to appropriately place bus stops within coverage areas that meet regulatory requirements. At the same time, the entrances and exits of bus stops should be reasonably laid out to improve accessibility for nearby residents. For those living farther from stops, shuttle buses should be set up to reduce their travel time to the stops, and additional bike-sharing points should be established around the stops to promote non-motorized travel. For sections lacking bay-style designs, it is necessary to implement appropriate platform-style bay modifications based on the specific layout of bus stops and the actual conditions of surrounding roads to make bus travel more user-friendly, thereby promoting non-motorized travel and increasing the frequency of green transportation.

## 5. Conclusion

To explore the nonlinear relationship and effective threshold range between the built environment and the efficiency of non-motorized travel in the central urban area of Lanzhou City, this study utilizes various open-source urban data, such as OpenStreetMap and Points of Interest (POI), in conjunction with the XGBoost model to predict and explain the nonlinear relationships between various built environment factors and non-motorized travel efficiency. XGBoost is a machine learning method that does not assume a specific function (such as linear or logarithmic relationships) a priori, allowing for accurate predictions of the nonlinear relationships among variables related to the research subject. The results also confirm that the performance of the XGBoost model significantly surpasses that of traditional linear regression models. This study employs five types of explanatory variables: point features, line features, area features, spatial features, and organizational features, selecting relevant representative variables within each feature type. The findings indicate that the branch road/street, land-use mix, land-use density, neighborhood entrance/exit density, bus station density, and dead-end-roads density are key factors influencing non-motorized travel efficiency, accounting for a total relative importance of 81.37%. Furthermore, the study reveals that each factor exists within an effective influence threshold range. The results contribute to a better understanding of how various built environment variables affect the efficiency of non-motorized travel, providing planners and decision-makers with insights into effective thresholds and policy implications. By formulating targeted environmental intervention measures, the overall operational efficiency of urban traffic microcirculation can be enhanced. Based on the above conclusions, targeted pedestrian optimization strategies for the core area of Chengguan District in Lanzhou City are proposed from three aspects: promoting a small-scale road network pattern, appropriate land-use mix, and enhancing public transport friendliness. The aim is to provide references and insights for similar regions in terms of pedestrian optimization construction and promoting the physical and mental health of residents.

This study also has certain limitations. Firstly, it only considers two states—workdays and weekends—along with peak hours (7:00–9:00, 17:00–19:00) and non-peak hours (9:00–17:00) for non-motorized travel time data. Future research could analyze the impact of different holidays (e.g., Spring Festival, National Day) on non-motorized travel efficiency. Secondly, this study focuses solely on the efficiency of non-motorized travel; future research could include analyses of motor vehicle and pedestrian movements for a more comprehensive evaluation of overall traffic microcirculation. Thirdly, the selection of variables in this study did not incorporate social variables such as population and economic factors. However, this does not imply that social variables do not have an impact on non-motorized travel efficiency. Future research could conduct a more detailed analysis in this regard.

## References

[1] Tao T, Næss P. Exploring nonlinear built environment effects on driving with a mixed-methods approach. Transportation Research Part D: Transport and Environment. 2022;111. doi: 10.1016/j.trd.2022.103443

[2] Sung H, Choi K, Lee S, Cheon S. Exploring the impacts of land use by service coverage and station-level accessibility on rail transit ridership. Journal of Transport Geography. 2014;36:134–40. doi: 10.1016/j.jtrangeo.2014.03.013

[3] Li S, Lyu D, Liu X, Tan Z, Gao F, Huang G, et al. The varying patterns of rail transit ridership and their relationships with fine-scale built environment factors: Big data analytics from Guangzhou. Cities. 2020;99. doi: 10.1016/j.cities.2019.102580

[4] Zhao Y. From planning to market: The shift of the road-landuse mode. City Planning Review. 2002;(10):24–30.

[5] Luo Q, Yang J, Wu W, Liu G. Designing microcirculatory road network for open block system. Journal of Transportation Systems Engineering and Information Technology. 2019;19(03):195–201. doi: 10.16097/j.cnki.1009-6744.2019.03.029

[6] Li G. The improvement of multimodal microcirculation transportation in mediumsized and small cities: A case study of Lishui. Urban Planning Forum. 2019;(S1):241–8. doi: 10.16361/j.upf.201907029

[7] Li W, Kamargianni M. Providing quantified evidence to policy makers for promoting bike-sharing in heavily air-polluted cities: A mode choice model and policy simulation for Taiyuan-China. Transportation Research Part A: Policy and Practice. 2018;111:277–91. doi: 10.1016/j.tra.2018.01.019

[8] Wang L, Zhou K, Zhang S, Moudon AV, Wang J, Zhu Y-G, et al. Designing bike-friendly cities: Interactive effects of built environment factors on bike-sharing. Transportation Research Part D: Transport and Environment. 2023;117. doi: 10.1016/j.trd.2023.103670

[9] Ao Y, Zhang Y, Wang Y, Chen Y, Yang L. Influences of rural built environment on travel mode choice of rural residents: The case of rural Sichuan. Journal of Transport Geography. 2020;85. doi: 10.1016/j.jtrangeo.2020.102708

[10] Wang D, Cao X. Impacts of the built environment on activity-travel behavior: Are there differences between public and private housing residents in Hong Kong? Transportation Research Part A: Policy and Practice. 2017;103:25–35. doi: 10.1016/j.tra.2017.05.018

[11] Zhang S, Li J, Wang L, Kwan M-P, Chai Y, Du Y, et al. Examining the association between the built environment and active travel using GPS data: A study of a large residential area (Daju) in Shanghai. Health & Place. 2023;79. doi: 10.1016/j.healthplace.2023.102971 36682263

[12] Eggimann S. The potential of implementing superblocks for multifunctional street use in cities. Nature Sustainability. 2022;5(5):406–14. doi: 10.1038/s41893-022-00855-2 35614932 PMC7612763

[13] Liu J, Zhou J, Xiao L, Yang L. Effects of the built environment on pedestrian communing to work and school: The Hong Kong case, China. Progress in Geography. 2019;38(06):807–17.

[14] Cervero R, Sarmiento OL, Jacoby E, Gomez LF, Neiman A. Influences of Built Environments on Walking and Cycling: Lessons from Bogotá. International Journal of Sustainable Transportation. 2009;3(4):203–26. doi: 10.1080/15568310802178314

[15] Tu M, Li W, Orfila O, Li Y, Gruyer D. Exploring nonlinear effects of the built environment on ridesplitting: Evidence from Chengdu. Transportation Research Part D: Transport and Environment. 2021;93. doi: 10.1016/j.trd.2021.102776

[16] Luo X, Zhang W, Chai Y. Research on threshold effects of built environment settings in 15-minute life-circles. Geographical Research. 2022;41(08):2155–70.

[17] Aghaabbasi M, Chalermpong S. Machine learning techniques for evaluating the nonlinear link between built-environment characteristics and travel behaviors: A systematic review. Travel Behaviour and Society. 2023;33. doi: 10.1016/j.tbs.2023.100640

[18] Tong Z, An R, Liu Y. Impact of the built environment on residents’ commuting mode choices: A case study of urban village in Wuhan City. Progress in Geography. 2021;40(12):2048–60.

[19] Shi Z, Yan C, He M, Liu Y, He M. Non-linear effects of built environment on travel mode choices for elderly. Journal of Transportation Engineering and Information. 2023;21(01):49–63. doi: 10.19961/j.cnki.1672-4747.2022.06.027

[20] Chiu B-y. Relationship between motorcycle travel and the built environment: Evidence from Taipei, Taiwan. Journal of Transport Geography. 2023;110. doi: 10.1016/j.jtrangeo.2023.103607

[21] Ding C, Zhou X, Jason Cao X, Yang J. Spatial and mediation analysis of the influences of residential and workplace built environments on commuting by car. Transportation Research Part A: Policy and Practice. 2023;171. doi: 10.1016/j.tra.2023.103662

[22] Gao F, Yang L, Han C, Tang J, Li Z. A network-distance-based geographically weighted regression model to examine spatiotemporal effects of station-level built environments on metro ridership. Journal of Transport Geography. 2022;105. doi: 10.1016/j.jtrangeo.2022.103472

[23] Huang F, Tang J, Lin H, Han S, Zhao P. Built environment effects on the spatio-temporal distribution of shared bikes based on multi-scale geographic weighted regression. Geographical Research. 2023;42(09):2405–18.

[24] Teng L, Yang Y. The study on the traffic problems of valley-city: A case study of Lanzhou City. Economic Geography. 2002;(01):72–6.

[25] Aston L, Currie G, Kamruzzaman M, Delbosc A, Brands T, van Oort N, et al. Multi-city exploration of built environment and transit mode use: Comparison of Melbourne, Amsterdam and Boston. Journal of Transport Geography. 2021;95. doi: 10.1016/j.jtrangeo.2021.103136

[26] Ding C, Liu T, Cao X, Tian L. Illustrating nonlinear effects of built environment attributes on housing renters’ transit commuting. Transportation Research Part D: Transport and Environment. 2022;112. doi: 10.1016/j.trd.2022.103503

[27] Ewing R, Cervero R. Travel and the Built Environment. Journal of the American Planning Association. 2010;76(3):265–94. doi: 10.1080/01944361003766766

[28] Yu Z, Li P, Schwanen T, Zhao P, Zhao Z. Role of rural built environment in travel mode choice: Evidence from China. Transportation Research Part D: Transport and Environment. 2023;117. doi: 10.1016/j.trd.2023.103649

[29] Pan Y, He SY. An investigation into the impact of the built environment on the travel mobility gap using mobile phone data. Journal of Transport Geography. 2023;108. doi: 10.1016/j.jtrangeo.2023.103571

[30] Yang Y, Sasaki K, Cheng L, Liu X. Gender differences in active travel among older adults: Non-linear built environment insights. Transportation Research Part D: Transport and Environment. 2022;110. doi: 10.1016/j.trd.2022.103405

[31] Wang X, Huang D, Liu Y, Yao Y, Xiang L. Impact of human-scale street space quality on walking to school by school-age childred: A case study of Beijing. City Planning Review.1-12.

[32] Kamruzzaman M, Shatu FM, Hine J, Turrell G. Commuting mode choice in transit oriented development: Disentangling the effects of competitive neighbourhoods, travel attitudes, and self-selection. Transport Policy. 2015;42:187–96. doi: 10.1016/j.tranpol.2015.06.003

[33] Li K, Yang D. Influence of individual attribute and built environment on convenience of public transportation for elderly. Journal of Transportation Systems Engineering and Information Technology. 2023;23(02):161–7. doi: 10.16097/j.cnki.1009-6744.2023.02.017

[34] Ma J, Zhao F, Yin C, Tang W. Spatial-temporal heterogeneity effects of built environment and taxi demand on ride-hailing demand. Journal of Transportation Systems Engineering and Information Technology. 2023:1–13.

[35] Long X, Zhao H, Zhou M, Mao J, Chen Y. Spatiotemporal heterogeneity of the impact of built environment in Chengdu on online car-hailing passengers’ pick-up points. Scientia Geographica Sinica. 2022;42(12):2076–84. doi: 10.13249/j.cnki.sgs.2022.12.004

[36] Shao H, Jin C, Zhong Y, Mao W. The impact of HaiKou’s urban built environment on online car-hailing commuting during park hours: Based on didi travel data. Human Geography. 2022;37(05):130–9. doi: 10.13959/j.issn.1003-2398.2022.05.016

[37] Yu B, Liang Y, Yang L. Exploring the relationship between bike-sharing ridership and built environment characteristics: A case study based on GAMM in Boston. World Regional Studies. 2023;32(02):48–58.

[38] Wang Z, Li H, Liu Z. Influence analysis of urban built environment on the return quantity of shared bikes. Journal of Chongqing Jiaotong University (Natural Science). 2023;42(07):128–35+45.

[39] Yu C, Deng Y, Qin Z, Yang C, Yuan Q. Traffic volume and road network structure: Revealing transportation-related factors on PM2.5 concentrations. Transportation Research Part D: Transport and Environment. 2023;124. doi: 10.1016/j.trd.2023.103935

[40] Ji S, Heinen E, Wang Y. Non-linear effects of street patterns and land use on the bike-share usage. Transportation Research Part D: Transport and Environment. 2023;116. doi: 10.1016/j.trd.2023.103630

[41] Yang W. The nonlinear effects of multi-scale built environments on CO2 emissions from commuting. Transportation Research Part D: Transport and Environment. 2023;118. doi: 10.1016/j.trd.2023.103736

[42] Ding C, Cao X, Næss P. Applying gradient boosting decision trees to examine non-linear effects of the built environment on driving distance in Oslo. Transportation Research Part A: Policy and Practice. 2018;110:107–17. doi: 10.1016/j.tra.2018.02.009

[43] Yang H, Zhang Q, Helbich M, Lu Y, He D, Ettema D, et al. Examining non-linear associations between built environments around workplace and adults’ walking behaviour in Shanghai, China. Transportation Research Part A: Policy and Practice. 2022;155:234–46. doi: 10.1016/j.tra.2021.11.017

[44] Tao T, Cao J. Exploring nonlinear and collective influences of regional and local built environment characteristics on travel distances by mode. Journal of Transport Geography. 2023;109. doi: 10.1016/j.jtrangeo.2023.103599

[45] Yang H, Zheng R, Li X, Huo J, Yang L, Zhu T. Nonlinear and threshold effects of the built environment on e-scooter sharing ridership. Journal of Transport Geography. 2022;104. doi: 10.1016/j.jtrangeo.2022.103453

[46] Cui X, Yu B, Yang L, Liang Y, Zhang L, Fang H. Spatio-temporal characteristics and non-linear influencing factors of urbanrail transit: The case of Chengdu using the gradient boosting decision tree. Economic Geography. 2021;41(07):61–72. doi: 10.15957/j.cnki.jjdl.2021.07.007

[47] Wang N, Wu J, Li S, Wang H, Peng Z. Spatial features of urban vitality and the impact of built environment on them based on multi-source data: A case study of Shenzhen. Tropical Geography. 2021;41(06):1280–91. doi: 10.13284/j.cnki.rddl.003406

[48] Gao K, Yang Y, Gil J, Qu X. Data-driven interpretation on interactive and nonlinear effects of the correlated built environment on shared mobility. Journal of Transport Geography. 2023;110. doi: 10.1016/j.jtrangeo.2023.103604

[49] Ma S. Research on the relationship between urban built environment and bicycle usage: A case study of London and Shanghai [Master]: Huazhong University of Science and Technology; 2019.

[50] Chen E, Ye Z. Identifying the nonlinear relationship between free-floating bike sharing usage and built environment. Journal of Cleaner Production. 2021;280. doi: 10.1016/j.jclepro.2020.124281

[51] Liu Z, Liu J, Hu R, Yang B, Huang X, Yang L. Calendar events’ influence on the relationship between metro ridership and the built environment: A heterogeneous effect analysis in Shenzhen, China. Tunnelling and Underground Space Technology. 2023;141. doi: 10.1016/j.tust.2023.105388

[52] Thrane C. Examining tourists’ long-distance transportation mode choices using a Multinomial Logit regression model. Tourism Management Perspectives. 2015;15:115–21. doi: 10.1016/j.tmp.2014.10.004

[53] Bouscasse H, Joly I, Peyhardi J. A new family of qualitative choice models: An application of reference models to travel mode choice. Transportation Research Part B: Methodological. 2019;121:74–91. doi: 10.1016/j.trb.2018.12.010

[54] Liu M, Liu Y, Ye Y. Nonlinear effects of built environment features on metro ridership: An integrated exploration with machine learning considering spatial heterogeneity. Sustainable Cities and Society. 2023;95. doi: 10.1016/j.scs.2023.104613

[55] He P, Li W, Li Y, Xu Q. Spatial patterns of nonlinear effects of built environment on Beijing subway ridership. Journal of Transportation Systems Engineering and Information Technology. 2023;23(03):187–94. doi: 10.16097/j.cnki.1009-6744.2023.03.020

[56] Caigang Z, Shaoying L, Zhangzhi T, Feng G, Zhifeng W. Nonlinear and threshold effects of traffic condition and built environment on dockless bike sharing at street level. Journal of Transport Geography. 2022;102. doi: 10.1016/j.jtrangeo.2022.103375

[57] Wang Y, Li J, Su D, Zhou H. Spatial-temporal heterogeneity and built environment nonlinearity in inconsiderate parking of dockless bike-sharing. Transportation Research Part A: Policy and Practice. 2023;175. doi: 10.1016/j.tra.2023.103789

[58] Ji S, Wang X, Lyu T, Liu X, Wang Y, Heinen E, et al. Understanding cycling distance according to the prediction of the XGBoost and the interpretation of SHAP: A non-linear and interaction effect analysis. Journal of Transport Geography. 2022;103. doi: 10.1016/j.jtrangeo.2022.103414

[59] Shao Q, Zhang W, Cao X, Yang J. Nonlinear and interaction effects of land use and motorcycles/E-bikes on car ownership. Transportation Research Part D: Transport and Environment. 2022;102. doi: 10.1016/j.trd.2021.103115

[60] Wang T, Hu S, Jiang Y. Predicting shared-car use and examining nonlinear effects using gradient boosting regression trees. International Journal of Sustainable Transportation. 2020;15(12):893–907. doi: 10.1080/15568318.2020.1827316

[61] Chen C, Tang Y. Nonlinear effects of built environment on travel safety of the elderly pedestrians: A case in Yuzhong district, Chongqing. Science Technology and Engineering. 2023;23(16):7112–9.

[62] Yang H, Shen L, Hu Y, Zhang Y, Peng J. Spatial and temporal characteristics of elderly people’s metro travelbehavior and its non-linear relationship with the built environment: A case study of Wuhan City. Progress in Geography. 2023;42(03):491–504.

[63] Liu J, Xiao L, Zhou J, Guo Y, Yang L. Non-linear relationships between the built environment and walking to school: Applying extreme gradient boosting method. Progress in Geography. 2022;41(02):251–63.

[64] Fang G. Research on traffic congestion in river valley city based on resident travel survey: Taking Lanzhou city as an example. Communications Science and Technology Heilongjiang. 2020;43(05):230–1. doi: 10.16402/j.cnki.issn1008-3383.2020.05.127

[65] Wang Y, Wang Y. Research on evaluation and optimization of traffic micro-circulation in Suzhou ancient city. Shanghai Urban Planning Review. 2021;(06):113–9.

[66] Liu W. Micro transportation circulation and local street system research development. Planners. 2009;25(06):21–4.

[67] Jiang Y, Wang Y, Zhao B, Han P, Yao Z. Optimization design of urban traffic microcirculation network based on one-way traffic. China Transportation Review. 2019;41(01):65–70+126.

[68] Guo Y, Xia Z, Guo C. Operation efficiency and determinant factors of traffic microcirculation in urban central areas:A nonlinear analysis based on the XGBoost model. World Architecture. 2023;(07):22–3. doi: 10.16414/j.wa.2023.07.030

[69] Chen T, Guestrin C. XGBoost. Proceedings of the 22nd ACM SIGKDD International Conference on Knowledge Discovery and Data Mining2016. p. 785–94.

[70] Cao W, Liu Y, Mei H, Shang H, Yu Y. Short-term district power load self-prediction based on improved XGBoost model. Engineering Applications of Artificial Intelligence. 2023;126. doi: 10.1016/j.engappai.2023.106826

